# Inferring pathway dysregulation in cancers from multiple types of omic data

**DOI:** 10.1186/s13073-015-0189-4

**Published:** 2015-06-26

**Authors:** Shelley M MacNeil, William E Johnson, Dean Y Li, Stephen R Piccolo, Andrea H Bild

**Affiliations:** Department of Oncological Sciences, University of Utah, Salt Lake City, UT USA; Department of Pharmacology and Toxicology, University of Utah, Salt Lake City, UT USA; Division of Computational Biomedicine, Boston University School of Medicine, Boston, MA USA; Department of Medicine, University of Utah, Salt Lake City, UT USA; Department of Human Genetics, University of Utah, Salt Lake City, UT USA; Department of Biology, Brigham Young University, Provo, UT USA

## Abstract

Although in some cases individual genomic aberrations may drive disease development in isolation, a complex interplay among multiple aberrations is common. Accordingly, we developed Gene Set Omic Analysis (GSOA), a bioinformatics tool that can evaluate multiple types and combinations of omic data at the pathway level. GSOA uses machine learning to identify dysregulated pathways and improves upon other methods because of its ability to decipher complex, multigene patterns. We compare GSOA to alternative methods and demonstrate its ability to identify pathways known to play a role in various cancer phenotypes. Software implementing the GSOA method is freely available from https://bitbucket.org/srp33/gsoa.

**Electronic supplementary material:**

The online version of this article (doi:10.1186/s13073-015-0189-4) contains supplementary material, which is available to authorized users.

## Background

A pressing goal within the research community is to further elucidate cellular processes affected by molecular aberrations by better utilizing the wealth of genomic data available. Genomic aberrations that occur within tumors are notoriously heterogeneous - even within a given cancer type, aberrations occur in a wide variety of genes due to different mechanisms, including aberrant gene expression, somatic mutations, epigenetic changes, and DNA copy-number alterations [1]. However, even though the genomic landscapes of individual tumors vary, the same biological pathways are often affected across many tumors of the same type. For example, Wood *et al.* showed that p110α, the active component of PI3K, was mutated in 11.9 % of breast tumors; however, when other genes in the same biological pathway were considered, 33.3 % of tumors contained a mutation in the PI3K network and thus had potential to increase proliferation and suppress apoptosis [2]. Pathway-level aggregation can place such observations in biological context [2, 3]. In addition, pathway-based, targeted cancer therapies are more specific and can be less toxic than conventional chemotherapies [4]. Therefore, understanding the pathway activity that underlies specific cancers may lead to better treatments. Because one type of data alone may provide an incomplete view of pathway activity - and due to the availability of multi-omic data from projects such as The Cancer Genome Atlas (TCGA) [5] - there is a need to develop methods capable of analyzing multiple types of omic data and thus to provide a more comprehensive view of cancer at the pathway level.

Gene set analysis (GSA) methods are widely used to analyze biological data at the pathway level [6–10]. Gene Set Enrichment Analysis (GSEA) [3] is the most popular such method, and it has been extended and improved by many [11–13]. GSA methods differ in the ways they calculate gene-level statistics, derive null hypotheses, compute gene set statistics, and assess significance [9]. However, the primary goal of each of these methods is to map omic measurements to gene sets that represent logical groupings of genes, including biological processes, molecular functions, and cellular components. The primary output of these methods is a ranked list that indicates which gene sets are considered to be most significantly dysregulated between two conditions. This list may then be used to inform computational and/or bench research, which can then help to uncover the precise mechanisms underlying the biological phenomenon. These methods have been instrumental to important biological discoveries, such as the identification of genes involved in oxidative phosphorylation whose expression is correlated with diabetes [3], establishment of molecular subtypes in prostate cancer [14], and identification of pathways involved in glioblastoma survival [15].

Existing GSA methods have proven useful in analyzing gene expression data but suffer from various limitations. Most methods are designed to evaluate only one type of omic data at a time. Although many GSA methods are designed to analyze microarray data [3, 11, 16–19], relatively few methods are capable of analyzing RNA-Sequencing data [20–23], and even fewer handle single-nucleotide variant data [19, 24, 25] or DNA methylation data [26]. Second, few existing methods account for intervariable dependencies. Taking into account such dependencies is critical because molecular-level interactions occur ubiquitously within cells. In addition, many methods do not consider the directionality of gene changes, even though pathway dysregulation may result from up- and downregulation of genes.

To address these issues, we have developed a novel approach, Gene Set Omic Analysis (GSOA). Under the assumption that aberrant biological activity is reflected in omic measurements from multiple data types, GSOA seeks to identify multi-gene patterns that differ between biological samples representing two conditions. This approach is based on the premise that a given gene typically influences a biological process in conjunction with other gene(s) and that genes affecting the process may differ considerably from sample to sample. Accordingly, individual genes may show no statistical significance in isolation; however, multi-gene patterns may reflect these dynamics. The GSOA method employs the Support Vector Machines algorithm [27], which is designed to account for complex dependencies among variables (in this case, genes). When such patterns can be identified consistently for a given gene set, that gene set is hypothesized to play a role in the condition of interest. GSOA can be applied to any type of omic data for which gene set annotations exist; this includes (but is not limited to) gene-expression microarray data, RNA-Sequencing data, single-nucleotide variant data (SNV), DNA copy-number variation data (CNV), and epigenetic data.

We have validated GSOA using simulated data, gene-expression microarray data, RNA-sequencing data, CNV data, somatic SNV data, and combinations of these data types. Using data from hundreds of tumors in TCGA, we have identified pathways that show patterns of dysregulation between HER2-positive and HER2-negative breast tumors and pathways whose expression differs between individuals who carry a somatic mutation in the RAS subfamily and those who do not. Additionally, we have compared uterine serous carcinomas (USC) against uterine endometrioid carcinomas (UEC) and have identified pathways that may play a role in USC treatment resistance. GSOA suggests that the MYC pathway plays an important role in USC tumors. Further analysis of gene expression levels and somatic mutations in these tumors suggests that key proteins in the MYC pathway are upregulated in USC tumors; this finding has clinical implications and provides motivation for more in-depth biological examination into this mechanism. Our approach serves as a way to extract biologically relevant patterns from large, heterogeneous, omic datasets in support of subsequent, hypothesis-driven experimental studies.

## Methods

### Software implementation

**Fig. 1:**
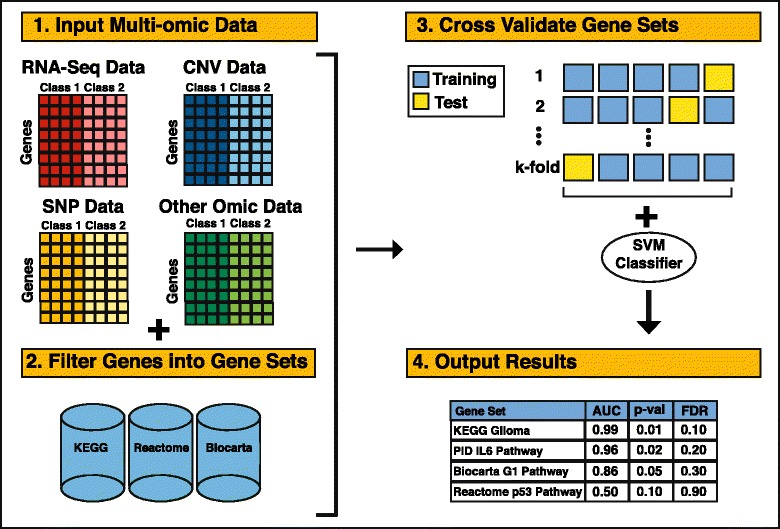
High-level description of the GSOA methodology. After mapping input data to gene sets, GSOA uses the SVM algorithm to assess how accurately samples from the two classes can be classified. Gene sets for which relatively high classification accuracy is attained are considered most likely to play a role in the biological question of interest

The GSOA code implementation is freely available at [28]. A schematic overview of the GSOA method is shown in Fig. 1. Required inputs are: (1) a data file containing omic measurements for each sample; (2) a data file indicating the condition or phenotype status for each sample; and (3) a file that indicates which genes map to which gene sets. Data file #1 uses a simple matrix format in which samples represent columns and rows represent genomic features. This file also should contain a header row with an identifier for each sample. Each row should start with a value that indicates the gene name. Multiple rows per gene may be listed - for example, when an omic-profiling technology produces multiple data values per gene. When multiple types of omic data are available for the same samples, multiple data files can be specified using wildcards. Data file #2 contains two columns; the first value in each row should be a sample identifier (and should correspond exactly with the identifiers in data file #1), and the second value should indicate which class (for example, condition or phenotype status) the sample represents. Data file #3 should be in Gene Matrix Transposed (GMT) format as used in the Molecular Signatures Database [29]. The first value in each row is the gene set name, the second value is a descriptor, and the remaining, tab-separated values are the genes associated with that gene set. Data files #2 and #3 should contain no header row, and all files should use tab characters as delimiters. Our software implementation of GSOA provides examples of each of these file types.

### Algorithm

For each gene set, the GSOA algorithm performs the following steps in sequence: (1) the omic data are filtered to include only the genes that belong to that gene set; (2) a classification algorithm predicts the class of each sample via k-fold cross validation; and (3) the area under the receiver operating characteristic curve (AUC) is calculated as a measure of prediction accuracy. Prior to classification, we mean center the data and scale it to unit variance; however, we recommend that omic data also be preprocessed (for example, background corrected) using methodologies appropriate for a given omic-profiling technology. For step #2, we use five cross-validation folds by default; the user can specify alternate values for *k*. Any classification algorithm could be used for step #2; however, we use the Support Vector Machines (SVM) algorithm because it is designed to account for complex dependencies in high-dimensional data and has been shown to perform consistently well compared to other classification algorithms [30]. We use the radial basis function SVM kernel with default parameters as implemented in the *scikit-learn* framework [31], which uses LibSVM [32]; it is also possible to specify alternate values for the cost and gamma parameters. In addition, we provide an option for users to auto-tune the SVM parameters via nested cross validation.

When multiple types of omic data are used as input, GSOA merges the data, and the classification algorithm builds a single SVM model that integrates data across the omic types. In deriving these integrated models, GSOA includes whichever genes map to a given pathway for each omic type, even though different omic technologies may profile different genes. However, GSOA only considers samples that contain data for all omic types.

For a given gene set, a relatively high AUC score (maximum of 1.0) indicates that the algorithm accurately predicted the group to which each sample belongs. An AUC value near 0.5 indicates that the predictions performed no better than would be expected if the samples were assigned randomly to either group.

To remove any correlation between gene-set size and AUC values, we incorporated a step into our algorithm that repeats cross-validation for randomly selected gene sets. The number of genes in each random gene set corresponds to the sizes of the actual gene sets; however, to reduce computational burden, we use random gene sets of pre-specified sizes (1, 5, 10, 25, 50, 75, 100, 125, 150, 200, 250, 300, 400, 500+) that correspond to the (rounded up) sizes of the actual gene sets. For example, if the actual gene sets had 8, 47, 99, 232, and 245 genes, respectively, the random gene sets would contain 10, 50, 100, and 250 genes. After performing cross-validation repeatedly (100 times by default) for each random gene set size, the resulting AUC values represent a null distribution. For each actual gene set, we calculate an empirical *P* value as the fraction of AUC values from the corresponding null distribution that exceed the actual AUC value. This approach generates a *P* value that is independent of pathway size (see Results). GSOA produces a rank-ordered list that indicates the AUC, *P* value, and Benjamini-Hochberg false discovery rate (FDR) for each gene set [33].

## Results

Researchers often desire to characterize the signaling pathways that play important roles in a particular phenotype. A common approach is to profile biological samples using one or more omic technologies and then to search for differences in measurements between the sample groups. Often these investigations are conducted at the individual gene level; however, such approaches may fail to account for cooperation among genes. We have developed the GSOA method, which seeks to identify multi-gene patterns that differ between biological samples from either of two groups. When such patterns can be identified for a particular gene set - for example, genes that participate in a given biological process - we assume that the genes play a coordinate role in the biomedical phenomenon of interest. We prioritize the gene sets according to how accurately biological samples from the two groups can be distinguished from each other, using only omic data for a given gene set. Unlike many existing approaches that identify gene sets that are either up- or downregulated as a whole, our method assumes that some genes will be upregulated and some will be downregulated and that these responses may vary across the samples. We use a machine-learning algorithm to identify complex, multidirectional patterns that differ between the two conditions. Table 1 lists the various datasets we used in our analyses.

**Table 1:** Number of samples contributing to each class and omic type for each dataset

Analysis	Class 1	Class 2	Somatic mutation	RNA-Seq	CNV	Microarray
p53 mutation status	17	33	-	-	-	50
	Wild-type	p53-mutated				
Gender	15	17	-	-	-	32
	Male	Female				
*RAS* mutation status (TCGA LUAD)	66	161	-	169	-	-
	Wild-type	RAS-mutated				
HER2 analysis (TCGA breast)	58	489	506	508	308	519
	HER2 +	Other breast				
USC analysis (TCGA endometrial)	53 USC	307 UEC	244	323	353	-

LUAD, lung adenocarcinoma; UCS, uterine serous carcinoma; UES, uterine endometrioid carcinoma

In a demonstrative example comparing breast-cancer subtypes, we observed that gene sets containing a relatively large number of genes resulted in higher overall AUC values (Additional file 1: Fig. S1A, Spearman correlation coefficient = 0.764). However our random-selection procedure for generating *P* values accurately corrects the *P* values for this bias (see Software implementation). Additional file 1: Fig. S1B shows that the resulting empirical *P* values - which indicate how likely one would observe a particular AUC value relative to randomly selected gene sets of similar size - show no bias toward larger gene sets.

### Validation using simulated data

We generated simulated data for 100 samples and 20,000 genes (see Additional file 1); in an initial evaluation, the samples were split evenly between two classes. We applied GSOA, GSEA [3], GAGE [20], and GSAA [19] to the simulated data and assessed how well each method predicted as significant the gene sets that contained signal genes (using FDR values as a metric). We compared GSOA against GSEA, GAGE, and GSAA because they are also supervised methods and are commonly used in the bioinformatics community. Like GSOA, GAGE and GSAA can be applied to multiple types of gene-expression data. In addition, GAGE can account for gene directionality. For gene sets containing a minimum of 10 signal genes, GSOA consistently produced FDR values below 0.20. In contrast, GSEA, GAGE, and GSAA produced FDR values below 0.20 for gene sets containing at least 15–25 signal genes (Additional file 1: Fig. S2). Accordingly, GSOA was more sensitive at identifying relatively subtle patterns within the data.

**Fig. 2:**
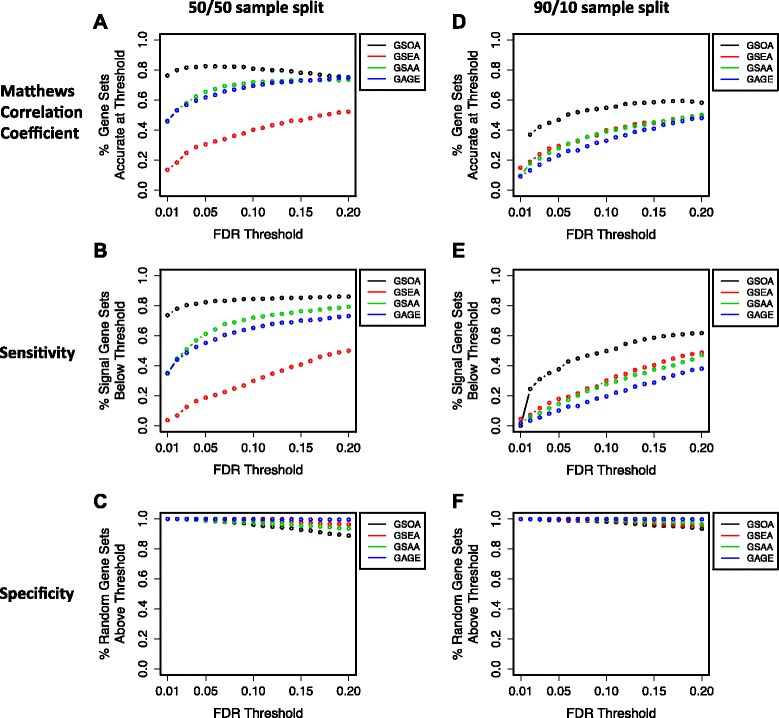
Results of cross-algorithm comparisons on simulated data. We compared GSOA against other methods using simulated data that contained interdependence among variables. For various FDR thresholds, we calculated the proportion of simulated gene sets containing signal that were considered significant and the proportion of gene sets containing only random data that were considered insignificant. Panels **a**-**c** show results for balanced data (50/50 sample split); Panels **d**-**f** show results for unbalanced data (90/10 sample split). See also Additional file 1: Fig. S4

Using the simulated data, we evaluated the balance between sensitivity and specificity for each method. In this context, sensitivity refers to an algorithm’s ability to identify as significant the gene sets that contained signal genes. Specificity refers to the algorithm’s ability to correctly classify (as insignificant) any gene set that contained no signal gene. We used the Matthews Correlation Coefficient (MCC) to quantify the balance between sensitivity and specificity [34]. For each gene set, the predictor was the FDR value that had been assigned to the gene set by each algorithm. Across all of the FDR thresholds that we tested, GSOA attained considerably higher MCC values than the competing methods (Fig. 2a). In particular, at relatively stringent FDR thresholds, as would be used in analyzing omic data, GSOA was much more sensitive than the other methods (Fig. 2b) and attained similar levels of specificity (Fig. 2c). For example, at an FDR threshold of 0.05, GSOA produced 243 (26 %) more true positives than GSAA, the best competing method (Additional file 1: Table S1A). GSOA produced 11 false positives (1 % of all signal gene sets), which was only three more than GSAA. At an FDR threshold of 0.20, GSOA and GAGE attained the same MCC value; GSOA produced 150 more true positives than GAGE, whereas GAGE produced 123 fewer false positives (Additional file 1: Table S1B).

As a follow-up analysis, we simulated a dataset in which 90 samples belonged to one class and 10 samples belonged to the other class, mimicking class imbalances that are common in omic studies. GSOA continued to perform best out of the methods, although the performance of all methods declined relative to the data that used a 50/50 class split (Fig. 2d-f, Additional file 1: Fig. S2).

We repeated these simulation analyses using *P* values rather than FDR values (Additional file 1: Figs. S3 and S4). The results were similar to when FDR values are used. Because 0.05 is an extremely common *P* value threshold, this was the maximum threshold we used in this part of the analysis.

For these analyses, we considered FDR and *P* value thresholds that are used in common research practice. Although GSOA performs better than (or at least similarly to) competing methods at these thresholds, it may not perform as well at less-stringent thresholds.

### Validation using benchmark microarray datasets

We analyzed GSOA’s ability to provide biologically meaningful results using microarray data from Subramanian *et al*. [3]. Again, we compared GSOA against GSEA, GAGE, and GSAA (see Additional file 1 for specific parameters). The p53 dataset contains gene expression values from 50 cancer cell lines that either harbored mutations in the *TP53* gene (33 cell lines) or were wild type (17 cell lines). This dataset has been used as a benchmark in numerous studies [3, 9, 18, 35]. p53 is a tumor suppressor protein involved in the cell cycle that induces apoptosis when a cell’s DNA becomes damaged [36]. In performing these comparisons, we used 522 canonical gene sets that were used in the original GSEA paper [3]. GSOA prioritized gene sets that are clearly related to p53 and cell-cycle function (see Table 2, Additional file 2). Refer to Additional file 1: Fig. S5 for the GSOA KEGG p53 pathway ROC curve. The other methods also performed well; however GSOA identified more gene sets that play a role in cell-cycle regulation.

**Table 2:** Validation and comparison to other methods in a p53 benchmark microarray dataset

	GSOA			GSEA			GAGE			GSAA		
Canonical gene sets	Rank	*P*	FDR	Rank	*P*	FDR	Rank	*P*	FDR	Rank	*P*	FDR
P53 pathway	1	0.001	0.037	1	0.000	0.009	26	0.093	0.822	1	0.000	0.566
P53 signaling	15	0.002	0.058	21	0.028	0.614	30	0.109	0.822	29	0.048	0.695
P53 hypoxia pathway	1	0.001	0.037	1	0.000	0.009	27	0.103	0.822	5	0.002	0.713
P53 up	1	0.001	0.037	1	0.000	0.065	20	0.083	0.822	1	0.000	0.595
DNA damage signaling	1	0.001	0.037	80	0.223	1	5	0.043	0.822	40	0.061	0.693
Radiation sensitivity	1	0.001	0.037	6	0.002	0.088	18	0.077	0.822	11	0.014	0.621
Cell cycle regulator	1	0.001	0.037	116	0.330	1	4	0.042	0.822	20	0.030	0.571
Cell cycle pathway	1	0.001	0.037	237	0.729	0.949	17	0.075	0.822	55	0.104	0.593
Cell cycle	15	0.002	0.058	172	0.531	0.930	7	0.046	0.822	93	0.175	1
Cell cycle arrest	43	0.021	0.255	166	0.509	0.887	41	0.141	0.822	216	0.396	1
Ras pathway	39	0.016	0.209	7	0.002	0.284	64	0.186	0.822	312	0.565	1
MAPK cascade	50	0.040	0.418	16	0.021	0.494	57	0.177	0.822	107	0.204	1
# of sig. gene sets	62			32			10			39		

Each method identified pathways related to p53 signaling and cell-cycle regulation. The ranks for these pathways were generally lower for GSOA than for the competing methods

We next tested each method using microarray data representing female and male lymphoblastoid cells using 522 canonical gene sets and 319 chromosomal gene sets, both of which were used in the original GSEA paper [3]. All methods performed well at prioritizing Y chromosome gene sets, which are likely to be differently regulated between male and female cells. Each method also identified gene sets associated with the X chromosome and sex-specific tissue; however, FDR values were highly variable across the methods (see Table 3, Additional file 2).

**Table 3:** Validation and comparison to other methods in a gender benchmark dataset

C1 cannonical gene sets (MSigDB)	GSOA			GSEA			GAGE			GSAA		
	Rank	*P*	FDR	Rank	*P*	FDR	Rank	*P*	FDR	Rank	*P*	FDR
chrY	1	0.001	0.079	1	0.000	0.000	1	0.001	0.297	1	0.000	0.105
chrYq11	1	0.001	0.079	1	0.000	0.000	2	0.002	0.335	1	0.000	0.105
chrYp11	1	0.001	0.079	1	0.000	0.002	6	0.052	0.923	1	0.000	0.210
chrXq26	17	0.035	0.623	114	0.652	0.961	316	0.979	0.979	284	0.892	0.959
chrXp22	156	0.505	0.985	4	0.002	1.000	3	0.008	0.895	1	0.000	1
# of sig. gene sets	29			7			6			21		
C2 cannonical gene sets (MsigDB)												
X-inactivation genes	17	0.031	0.770	1	0.000	0.000	2	0.008	0.914	1	0.000	0.135
Testis genes	71	0.127	0.885	1	0.000	0.067	3	0.008	0.914	1	0.000	0.890
GNF female genes	499	0.943	0.982	3	0.010	0.067	1	0.005	0.914	1	0.000	0.520
# of sig. gene sets	34			8			7			23		

We used the various methods to compare gene-expression levels between male and female cell lines

### Pathway-based comparison of lung adenocarcinoma samples based on RAS mutation status

Mutations in the RAS protein subfamily (*HRAS*, *NRAS*, *KRAS*) occur frequently in various types of cancer [37] and have a relatively high frequency in lung adenocarcinomas [38]. Oncogenic *RAS* mutations cause widespread changes in gene expression and lead to downstream activation of the PI3K/AKT and MAPK/ERK cascades, which increase cell growth and survival and causes changes in cellular differentiation [37]. RAS-driven cancers are extremely difficult to treat [37]. Identifying pathways activated by RAS mutations could help in developing targeted treatments for tumors with *RAS* mutations [39].

We applied GSOA, GSEA, GAGE, and GSAAseqSP [23] to RNA-Sequencing data from lung adenocarcinoma samples in TCGA (see Additional file 1 for specific parameters). We compared tumor samples in TCGA that contained a RAS subfamily mutation against samples that did not [40]. Previously, Bild *et al*. used experimental methods to identify genes dysregulated when RAS proteins are in an oncogenic state [41]. We evaluated whether GSOA could identify this gene set as significant in these tumor samples. As a control, we included 3,401 additional gene sets from the Molecular Signatures Database’s chemical and genetic perturbations collection [29]. GSOA successfully identified the RAS oncogenic gene set (*P* = 0.009) and identified fewer non-RAS related gene sets than the other methods (Additional file 1: Table S2, Additional file 3). Refer to Additional file 1: Fig. S6 for the Bild HRAS oncogenic signature gene set ROC curve). Such an analysis could also be applied to larger, curated gene set databases to aid in generating hypotheses about potential pathways to target in RAS-driven cancers.

### Comparison of HER2-positive and HER2-negative breast cancers using multiple types of omic data

**Fig. 3:**
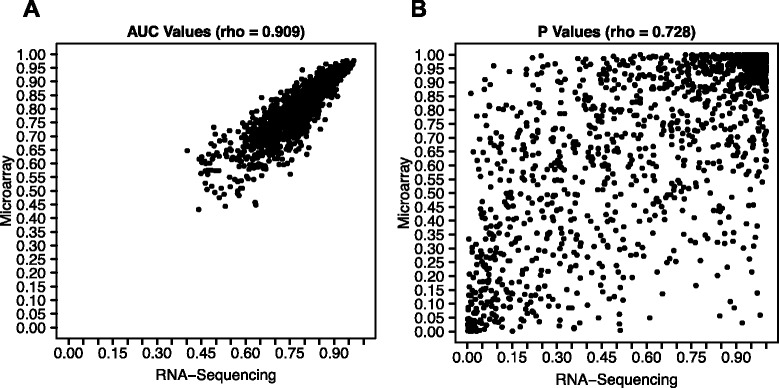
Correlation between GSOA output for microarray and RNA-Sequencing data. We used GSOA to compared HER2^+^ breast cancer samples against HER2^−^ samples from TCGA for either microarrays or RNA-Sequencing data. GSOA output values correlated strongly for **a**) AUC scores and **b**) *P* values. Spearman correlation coefficients were used to quantify similarity in ranks between the values

We sought to characterize pathway-level effects resulting from *HER2* amplification in breast tumors from TCGA [42]. We used GSOA to compare HER2 positive samples against HER2 negative samples (including normal controls). Using 1,320 canonical pathways [29], we first tested the robustness of our method to inter-platform differences by applying GSOA to microarray and RNA-Sequencing data from the same biological samples (see Additional file 1 for specific parameters). Although these technologies both measure RNA abundance, they produce data with different numerical distributions. The GSOA results for these two platforms were highly correlated (Spearman correlation coefficient = 0.909 for AUC values, 0.728 for *P* values, see Fig. 3). This level of correlation exceeds what we observed at the individual gene level (average correlation per gene = 0.676). Importantly, the findings for these two platforms led to similar biological conclusions. As expected, among the top results for both platforms were multiple pathways related to HER2 (ERBB2) signaling (see Additional file 4). Other top pathways included those related to PI3K signaling - which has been associated with the HER2 positive subtype [43].

**Fig. 4:**
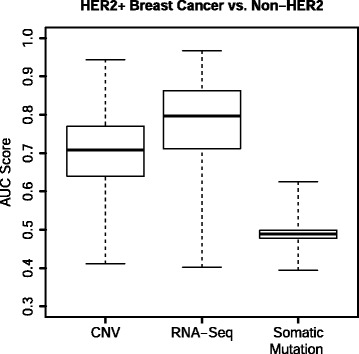
AUC scores for each omic type from the HER2 analysis. GSOA was applied to various types of omic data from TCGA. HER2^+^ breast cancer samples were compared against HER2^−^ samples. Predictions based on RNA-sequencing data attained the highest accuracy

We next applied GSOA to somatic CNV and SNV data for the same samples. RNA-Sequencing data yielded the highest AUC values overall (see Fig. 4). These findings are reasonable because the HER2-positive subtype is driven by *ERBB2* amplification, which leads to increased expression of HER2 and likely other interacting molecules [44]. We then compared GSOA predictions from RNA-Sequencing data against predictions for the other data types. The RNA-Sequencing and CNV predictions were modestly correlated (Spearman correlation coefficient = 0.294, Additional file 1: Fig. S7A), while the correlation between RNA-Sequencing and somatic mutation predictions was not significant (see Additional file 1: Fig. S7B). These findings suggest that various types of omic data may provide complementary evidence regarding the factors that influence pathway activity.

To test whether combining omic data was informative, we aggregated multi-omic data using two different methods. First, we integrated data from all omic types into a single dataset and allowed the SVM classifier to account for dependencies among these data types. Second, we used GSOA to analyze each data type separately and then combined the results using a rank-based *P* value calculation [45]. Both methods performed well and identified an equal number of significant gene sets related to ERBB2/PI3K signaling (see Additional file 1: Tables S3 and S4, and Additional file 4). The integrative approach identified more gene sets related to fibroblast growth factor receptor (FGFR) signaling, which is amplified in many breast cancers [46] and has been linked to lapatinib resistance in HER2-positive breast cancer cells [47]. Together, these results show that summarizing multiple types of omic data at the pathway level can shed light on biological processes that play a role in specific cancer phenotypes, and that information can be aggregated usefully across independent profiling platforms.

### Identification of MYC pathway dysregulation in uterine serous carcinoma

Most molecular studies in endometrial cancer have focused on the most common form, uterine endometrioid carcinoma (UEC), which is primarily driven by *PTEN* loss and mutations in *FGFR2*, *ARID1A*, *CTNNB1*, *PIK3CA*, *PIK2R1*, and *KRAS* [48]. In contrast, uterine serous carcinomas (USC) are an extremely aggressive subtype of endometrial cancer with poorly defined molecular pathway activity. Although USCs comprise only about 10 % of endometrial cancer cases, they are responsible for almost half of endometrial cancer deaths [49]. USCs are usually metastatic and chemoresistant, with a 50–80 % recurrence rate and an 18-25 % 5-year survival rate [50, 51]. Limited studies have shown USC to contain mutations in *TP53*, *PI3KCA*, *FBXW7*, and *PPP2RIA*, and overexpression of *ERBB2* [52–54]. The poorer survival and therapy response rates in USC highlight the need for a deeper understanding of the pathways that influence USC development in order to identify more effective therapies.

Here we sought to identify pathway level differences between USC and UEC. We used GSOA to compare 53 USC and 307 UEC tumor samples from the TCGA endometrial carcinoma study [55]. We evaluated RNA-Sequencing, somatic mutation, and CNV data (see Additional file 1 for specific parameters). GSOA prioritized pathways known to be dysregulated in either USC or UEC, as well as various pathways associated with cancer development in general. GSOA identified 87 significant pathways (*P* ≤0.05) for RNA-Sequencing, 144 for somatic mutations, 56 for CNV data, and 139 pathways when evidence was combined across these data types (rank-based *P* value method) (see Additional file 1: Table S5, Additional file 5). Alternatively, when the omic data were combined into a single SVM classifier, 67 gene sets were significant (see Additional file 1: Table S6, Additional file 5).

Alterations in the PI3K pathway have been shown to occur in over 80 % of UEC tumors [56] but not as frequently in USC [55]. The rank-based method consistently prioritized PI3K gene sets; with the KEGG phosphatidylinositol signaling system gene set ranking first along with many additional PI3K/ERBB related gene sets (Additional file 1: Table S5). Two PTEN gene sets also obtained significance - PTEN loss leads to PI3K activation [56]. In addition, four p53 gene sets were significant, which is expected because somatic mutations in *TP53* occur in most USCs [57]. Various additional pathways that had previously been associated with these cancer types were also identified [58].

The ranked-based method prioritized both the PID MYC pathway (*P* = 0.008) and the PID MYC active pathway (*P* = 0.057). We took interest to this pathway because literature on MYC pathway dysregulation in endometrial cancer is limited. *MYC* is a proto-oncogene, which can lead to deregulation of many genes, cause cellular proliferation, and result in tumor formation [59]. Upregulation of *MYC* via FGF signaling has been reported in endometrial cancer cells [60], and *MYC* amplifications have been associated with earlier disease recurrence in endometrial adenocarcinoma patients [61]. TCGA also reported *MYC* amplifications in their high-copy number cluster, which included some serous-like tumors [55].

For validation, we asked whether GSOA could identify MYC pathway dysregulation in an independent endometrial cancer dataset. We compared 11 USC and 22 UEC patient tumors from Mhawech-Fauceglia *et al*. (Gene Expression Omnibus accession number: GSE24537) [62]. GSOA identified significant differences in expression for the PID MYC Repression Pathway (*P* = 0.008), although the specific pathways differed - perhaps due to the smaller size of this dataset (see Additional file 5).

**Fig. 5:**
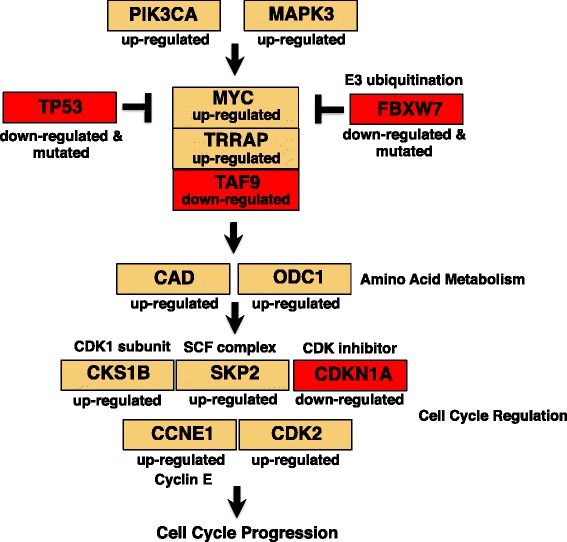
Summary of gene expression and somatic mutation differences between uterine serous (USC) and uterine endometrioid carcinomas (UEC) for the MYC pathway. Genes upregulated in USC were *MYC* (*P* = 3.3 × 10^−8^), *TRRAP* (*P* = 3.8 × 10^−4^), *PI3KCA* (*P* = 1.4 × 10^−10^), *MAPK3* (*P* = 5.8 × 10^−5^), *CAD* (*P* = 6.9 × 10^−9^), *ODC1* (*P* = 1.9 × 10^−11^), *CKS1B* (*P* = 9.2 × 10^−14^), *SKP2* (*P* = 3.0 × 10^−3^), *CDKN1A* (*P* = 6.8 × 10^−20^), *CCNE1* (*P* = 2.5 × 10^−18^), and *CDK2* (*P* = 2.0 × 10^−5^). *TP53* (*P* = 2.5 × 10^−3^) and *FBXW7* (*P* = 3.8 × 10^−4^) were upregulated and somatically mutated. *CDKN1A* (*P* = 6.8 × 10^−20^) and *TAF9* (5.9 × 10^−13^) were down in USC. This suggests upregulation of the MYC pathway in USC. A Wilcoxon rank test was used for RNA-Sequencing, and a Fisher’s Exact test for somatic mutations

To better understand why the MYC pathway was prioritized in our GSOA analyses, we investigated individual genes within this pathway as well as up- and downstream pathways. We compared gene expression levels and somatic mutation data for USC and UEC tumors and used the Wilcoxon rank test and Fisher’s exact test, respectively, to look for significant differences at the individual gene level (Additional file 1: Table S7). The modes of MYC dysregulation are highlighted in Fig. 5. Expression of *MYC* was elevated in USC (*P* = 3.3 × 10^−8^). MYC binding partner *TAF9* (*P* = 5.9 × 10^−13^) was down, and *TRRAP* (*P* = 3.8 × 10^−4^) was up. Downregulation of *TAF9* was unexpected, and may be worth further exploration. The MEK-ERK and PI3K pathways can induce *MYC* expression [59], and the *PIK3CA* (*P* = 1.4 × 10^−10^) and *MAPK3* (*P* = 5.8 × 10^−5^) genes were upregulated in USC, which we also saw in our GSOA analyses. Furthermore, we saw somatic mutations and downregulation of genes that negatively regulate MYC in USC, including *TP53* [63] (*P* = 2.5 × 10^−3^) and *FBXW7* (*P* = 3.8 × 10^−4^), which aids in MYC regulation via ubiquitination [64]. *FBXW7* mutations are common in USC [54], and also have been shown to increase MYC signaling in gastric cancers [65].

MYC is a master regulator of cellular proliferation via activation of nucleotide metabolism and cell cycle proteins [66]. We observed upregulation of genes known to be MYC targets that are involved in nucleotide/amino acid metabolism *CAD* (*P* = 6.9 × 10^−9^) and *ODC1* (*P* = 1.9 × 10^−11^). Many genes that promote the cell cycle and that are known to be regulated by MYC were upregulated in USC; these included *CKS1B* (*P* = 9.2 × 10^−14^), *SKP2* (*P* = 3.0 × 10^−3^), *CCNE1* (*P* = 2.5 × 10^−18^), and *CDK2* (*P* = 2.0 × 10^−5^). We also saw downregulation of *CDKN1A* (*P* = 6.8 × 10^−20^), a cell cycle inhibitor. Together, these results suggest that MYC is dysregulated in USC and highlight the potential importance of MYC targeted therapy for this cancer type.

## Discussion

Pathway-based analyses have become popular for providing insight into difficult-to-interpret omic data [6]. GSOA is a novel bioinformatics tool that can integrate data from multiple omic platforms at the pathway level to generate hypotheses about pathways that behave differently between biological conditions. Pathway-based approaches are particularly important for cancer interrogation because treatment modalities are moving towards targeting specific pathways. Therefore, an understanding of pathway dysregulation is a key step in developing personalized cancer care.

Our method builds upon a method developed by Pang *et al*. [67], which applied machine learning algorithms to gene-expression data to model dependencies among genes and ranked the results by prediction accuracy. Unlike their method, our approach can process multiple types of omic data, integrate data across multiple omic types, account for gene set size, and correct for class imbalances.

The ability to analyze omic data from various omic-profiling platforms is important when analyzing cancer data due to the compound effects of many types of alteration, including gene expression changes, copy-number variation, and single-nucleotide variants. This approach can also be applied to DNA methylation data, miRNA data, and proteomic data, as long as the features can be mapped to gene sets. Our analysis of HER2 pathway activity in HER2-positive breast tumors illustrates how integration of multi-omic data can identify gene sets that may be missed if analyzed separately. For example, a particular gene set may be borderline significant for individual types of omic data and thus go unnoticed; however, when the data are integrated, the gene set may reach significance.

One alternative approach that has been used commonly is over-representation analysis [6]. Such methods require a list of genes that are differentially expressed between two conditions and then prioritize gene sets in which these genes are enriched [68–70]. The simplicity of this approach could be seen as an advantage. However, over-representation methods treat each gene equally and independently, even though the magnitude of expression may differ considerably among the genes and dependencies may exist between genes. In contrast, an advantage of GSOA is that it examines omic data directly; thus it can account for (potentially) subtle differences in omic measurements that may span multiple genes.

We note that the biological relevance of GSOA results depends on the validity and relevance of the gene set annotations used as input. Although curated gene sets provide great breadth, they may be less precise than gene sets based on experimental observation. In addition, there is considerable overlap among gene sets described in multiple pathway resources. This redundancy complicates interpretation of results; however, when multiple pathways related to a given biological process are consistently prioritized by GSOA, this is an indication that the results are robust. In this paper, we have focused on pathways that show consistent significance in our analyses. It is also important to note that GSOA does not infer whether a given pathway is up- or downregulated as a whole; rather it assumes that when a pathway is dysregulated, some genes within the pathway may be upregulated while others are downregulated. Pathways that GSOA identifies as being dysregulated may serve as candidates for future mechanistic and functional studies, which can better dissect the contributions of individual genes.

## Conclusion

In summary, we have used our novel computational approach, GSOA, to identify signaling events with a known association among tumor subtypes to test the validity of our method. Results from these analyses highlight the power of our approach to accurately identify biological signal within omic data. Importantly, we have also used this approach to propose alternative pathways that influence development of specific cancer subtypes. For example, we propose that dysregulation of the critical master regulator MYC in uterine serous carcinomas may lead to treatment resistance. Such approaches are invaluable in our quest to distill large, heterogeneous, multi-omic data down to a form that leads to a better understanding of how disease develops and how it might be treated more effectively.

## Additional files

Additional file 1:
**Supplementary Methods, Figs. S1–S7, and Tables S1–S7.**
Additional file 2:
**Excel document containing the raw GSOA, GAGE, GSEA, and GSAA results for the p53 and gender microarray analyses.**
Additional file 3:
**Excel document that contains the raw GSOA, GAGE, GSEA, and GSAAseqSP results for the RAS mutation analysis in lung adenocarcinoma.**
Additional file 4:
**Excel document that contains the raw GSOA results for the HER2 analysis for RNA-Sequencing, microarray, somatic mutation, copy-number variation, and the rank-based and multi-omic analyses.**
Additional file 5:
**Excel document that contains the raw GSOA results from the TCGA endometrial cancer analyses for RNA-Sequencing, somatic mutation, copy-number variation, and the rank-based multi-omic analyses results.** It also contains the raw GSOA results for the GSE24537 microarray analysis.

## References

[1] Hanahan D, Weinberg RA (2011). Hallmarks of cancer: the next generation. Cell..

[2] Wood LD, Parsons DW, Jones S, Lin J, Sjöblom T, Leary RJ (2007). The genomic landscapes of human breast and colorectal cancers. Science..

[3] Subramanian A, Tamayo P, Mootha VK, Mukherjee S, Ebert BL, Gillette MA (2005). Gene set enrichment analysis: a knowledge-based approach for interpreting genome-wide expression profiles. Proc Natl Acad Sci U S A..

[4] Faivre S, Djelloul S, Raymond E (2006). New paradigms in anticancer therapy: targeting multiple signaling pathways with kinase inhibitors. Semin Oncol..

[5] Weinstein JN, Collisson EA, Mills GB, Shaw KRM, Ozenberger BA, Ellrott K (2013). The cancer genome atlas pan-cancer analysis project. Nat Genet..

[6] Khatri P, Sirota M, Butte AJ (2012). Ten years of pathway analysis: current approaches and outstanding challenges. PLoS Comput Biol..

[7] Hung J-H, Yang T-H, Hu Z, Weng Z, DeLisi C (2012). Gene set enrichment analysis: performance evaluation and usage guidelines. Brief Bioinform..

[8] Tarca AL, Bhatti G, Romero R (2013). A comparison of gene set analysis methods in terms of sensitivity, prioritization and specificity. PLoS One..

[9] Ackermann M, Strimmer K (2009). A general modular framework for gene set enrichment analysis. BMC Bioinf..

[10] Liu Q, Dinu I, Adewale AJ, Potter JD, Yasui Y (2007). Comparative evaluation of gene-set analysis methods. BMC Bioinf..

[11] Kim S-Y, Volsky DJ (2005). PAGE: parametric analysis of gene set enrichment. BMC Bioinf..

[12] Jiang Z, Gentleman R (2007). Extensions to gene set enrichment. Bioinformatics..

[13] Tian L, Greenberg SA, Kong SW, Altschuler J, Kohane IS, Park PJ (2005). Discovering statistically significant pathways in expression profiling studies. Proc Natl Acad Sci U S A..

[14] Markert EK, Mizuno H, Vazquez A, Levine AJ (2011). Molecular classification of prostate cancer using curated expression signatures. Proc Natl Acad Sci U S A..

[15] Tyekucheva S, Marchionni L, Karchin R, Parmigiani G (2011). Integrating diverse genomic data using gene sets. Genome Biol..

[16] Boorsma A, Foat BC, Vis D, Klis F, Bussemaker HJ (2005). T-profiler: scoring the activity of predefined groups of genes using gene expression data. Nucleic Acids Res..

[17] Wu D, Lim E, Vaillant F, Asselin-Labat M-L, Visvader JE, Smyth GK (2010). ROAST: rotation gene set tests for complex microarray experiments. Bioinformatics..

[18] Dinu I, Potter JD, Mueller T, Liu Q, Adewale AJ, Jhangri GS (2007). Improving gene set analysis of microarray data by SAM-GS. BMC Bioinf..

[19] Xiong Q, Ancona N, Hauser ER, Mukherjee S, Furey TS (2012). Integrating genetic and gene expression evidence into genome-wide association analysis of gene sets. Genome Res..

[20] Luo W, Friedman MS, Shedden K, Hankenson KD, Woolf PJ (2009). GAGE: generally applicable gene set enrichment for pathway analysis. BMC Bioinf..

[21] Hänzelmann S, Castelo R, Guinney J (2013). GSVA: gene set variation analysis for microarray and RNA-seq data. BMC Bioinf..

[22] Wang X, Cairns MJ (2014). SeqGSEA: a Bioconductor package for gene set enrichment analysis of RNA-Seq data integrating differential expression and splicing. Bioinformatics..

[23] Xiong Q, Mukherjee S, Furey TS (2014). GSAASeqSP: a toolset for gene set association analysis of RNA-Seq data. Sci Rep..

[24] Holden M, Deng S, Wojnowski L, Kulle B (2008). GSEA-SNP: applying gene set enrichment analysis to SNP data from genome-wide association studies. Bioinformatics..

[25] Zhang K, Cui S, Chang S, Zhang L, Wang J (2010). i-GSEA4GWAS: a web server for identification of pathways/gene sets associated with traits by applying an improved gene set enrichment analysis to genome-wide association study. Nucleic Acids Res.

[26] Geeleher P, Hartnett L, Egan LJ, Golden A, Raja Ali RA, Seoighe C (2013). Gene-set analysis is severely biased when applied to genome-wide methylation data. Bioinformatics..

[27] Vapnik VN (1999). An overview of statistical learning theory. IEEE Trans Neural Netw..

[28] Source code repository for Gene Set Omic Analysis software. Available at: https://bitbucket.org/srp33/gsoa

[29] Liberzon A, Subramanian A, Pinchback R, Thorvaldsdóttir H, Tamayo P, Mesirov JP (2011). Molecular signatures database (MSigDB) 3.0. Bioinformatics.

[30] Caruana R, Niculescu-Mizil A (2006). An empirical comparison of supervised learning algorithms. Proceedings of the 23rd international conference on Machine learning - ICML ’06.

[31] Pedregosa F, Varoquaux G, Gramfort A, Michel V, Thirion B, Grisel O (2011). Scikit-learn: Machine Learning in Python. J Mach Learn Res..

[32] Chang C-C, Lin C-J (2011). LIBSVM. ACM Trans Intell Syst Technol..

[33] Controlling the False Discovery Rate: A Practical and Powerful Approach to Multiple Testing on JSTOR. Available at: http://www.jstor.org/stable/2346101?seq=1#page_scan_tab_contents.

[34] Matthews BW (1975). Comparison of the predicted and observed secondary structure of T4 phage lysozyme. Biochim Biophys Acta - Protein Struct..

[35] Hua J, Bittner ML, Dougherty ER (2014). Evaluating gene set enrichment analysis via a hybrid data model. Cancer Inform..

[36] Freed-Pastor WA, Prives C (2012). Mutant p53: one name, many proteins. Genes Dev..

[37] Stephen AG, Esposito D, Bagni RK, McCormick F (2014). Dragging ras back in the ring. Cancer Cell..

[38] Suda K, Tomizawa K, Mitsudomi T (2010). Biological and clinical significance of KRAS mutations in lung cancer: an oncogenic driver that contrasts with EGFR mutation. Cancer Metastasis Rev..

[39] El-Chaar NN, Piccolo SR, Boucher KM, Cohen AL, Chang JT, Moos PJ (2014). Genomic classification of the RAS network identifies a personalized treatment strategy for lung cancer. Mol Oncol..

[40] Collisson EA, Campbell JD, Brooks AN, Berger AH, Lee W, Chmielecki J (2014). Comprehensive molecular profiling of lung adenocarcinoma. Nature..

[41] Bild AH, Yao G, Chang JT, Wang Q, Potti A, Chasse D (2006). Oncogenic pathway signatures in human cancers as a guide to targeted therapies. Nature..

[42] The Cancer Genome Atlas Network (2012). Comprehensive molecular portraits of human breast tumours. Nature.

[43] Kümler I, Tuxen MK, Nielsen DL (2014). A systematic review of dual targeting in HER2-positive breast cancer. Cancer Treat Rev..

[44] Perou CM, Sørlie T, Eisen MB, van de Rijn M, Jeffrey SS, Rees CA (2000). Molecular portraits of human breast tumours. Nature..

[45] Langfelder P, Horvath S (2008). WGCNA: an R package for weighted correlation network analysis. BMC Bioinf..

[46] Elbauomy Elsheikh S, Green AR, Lambros MBK, Turner NC, Grainge MJ, Powe D (2007). FGFR1 amplification in breast carcinomas: a chromogenic in situ hybridisation analysis. Breast Cancer Res..

[47] Azuma K, Tsurutani J, Sakai K, Kaneda H, Fujisaka Y, Takeda M (2011). Switching addictions between HER2 and FGFR2 in HER2-positive breast tumor cells: FGFR2 as a potential target for salvage after lapatinib failure. Biochem Biophys Res Commun..

[48] McConechy MK, Ding J, Cheang MCU, Wiegand KC, Senz J, Tone AA (2012). Use of mutation profiles to refine the classification of endometrial carcinomas. J Pathol..

[49] Hamilton CA, Cheung MK, Osann K, Chen L, Teng NN, Longacre TA (2006). Uterine papillary serous and clear cell carcinomas predict for poorer survival compared to grade 3 endometrioid corpus cancers. Br J Cancer..

[50] Del Carmen MG, Birrer M, Schorge JO (2012). Uterine papillary serous cancer: a review of the literature. Gynecol Oncol..

[51] El-Sahwi KS, Schwartz PE, Santin AD (2012). Development of targeted therapy in uterine serous carcinoma, a biologically aggressive variant of endometrial cancer. Expert Rev Anticancer Ther..

[52] Santin AD, Bellone S, Van Stedum S, Bushen W, Palmieri M, Siegel ER (2005). Amplification of c-erbB2 oncogene: a major prognostic indicator in uterine serous papillary carcinoma. Cancer..

[53] Kuhn E, Wu R-C, Guan B, Wu G, Zhang J, Wang Y (2012). Identification of molecular pathway aberrations in uterine serous carcinoma by genome-wide analyses. J Natl Cancer Inst..

[54] Le Gallo M, O’Hara AJ, Rudd ML, Urick ME, Hansen NF, O’Neil NJ (2012). Exome sequencing of serous endometrial tumors identifies recurrent somatic mutations in chromatin-remodeling and ubiquitin ligase complex genes. Nat Genet..

[55] The Cancer Genome Atlas Network (2013). Integrated genomic characterization of endometrial carcinoma. Nature.

[56] Cheung LWT, Hennessy BT, Li J, Yu S, Myers AP, Djordjevic B (2011). High frequency of PIK3R1 and PIK3R2 mutations in endometrial cancer elucidates a novel mechanism for regulation of PTEN protein stability. Cancer Discov..

[57] Acharya S, Hensley ML, Montag AC, Fleming GF (2005). Rare uterine cancers. Lancet Oncol..

[58] Szabó I, Kiss A, Schaff Z, Sobel G (2009). Claudins as diagnostic and prognostic markers in gynecological cancer. Histol Histopathol..

[59] Dang CV (2013). MYC, metabolism, cell growth, and tumorigenesis. Cold Spring Harb Perspect Med..

[60] Taniguchi F, Harada T, Sakamoto Y, Yamauchi N, Yoshida S, Iwabe T (2003). Activation of mitogen-activated protein kinase pathway by keratinocyte growth factor or fibroblast growth factor-10 promotes cell proliferation in human endometrial carcinoma cells. J Clin Endocrinol Metab..

[61] Borst MP, Baker VV, Dixon D, Hatch KD, Shingleton HM, Miller DM (1990). Oncogene alterations in endometrial carcinoma. Gynecol Oncol..

[62] Mhawech-Fauceglia P, Wang D, Kesterson J, Syriac S, Clark K, Frederick PJ (2011). Gene expression profiles in stage I uterine serous carcinoma in comparison to grade 3 and grade 1 stage I endometrioid adenocarcinoma. PLoS One..

[63] Kaddurah-Daouk R, Greene JM, Baldwin AS, Kingston RE (1987). Activation and repression of mammalian gene expression by the c-myc protein. Genes Dev..

[64] Nakayama KI, Nakayama K (2005). Regulation of the cell cycle by SCF-type ubiquitin ligases. Semin Cell Dev Biol..

[65] Calcagno DQ, Freitas VM, Leal MF, de Souza CRT, Demachki S, Montenegro R (2013). MYC, FBXW7 and TP53 copy number variation and expression in gastric cancer. BMC Gastroenterol..

[66] Van Dang C, McMahon SB (2010). Emerging concepts in the analysis of transcriptional targets of the MYC oncoprotein: are the targets targetable?. Genes Cancer..

[67] Pang H, Lin A, Holford M, Enerson BE, Lu B, Lawton MP (2006). Pathway analysis using random forests classification and regression. Bioinformatics..

[68] Thomas PD, Campbell MJ, Kejariwal A, Mi H, Karlak B, Daverman R (2003). PANTHER: a library of protein families and subfamilies indexed by function. Genome Res..

[69] Chang JT, Nevins JR (2006). GATHER: a systems approach to interpreting genomic signatures. Bioinformatics..

[70] Huang DW, Sherman BT, Tan Q, Kir J, Liu D, Bryant D (2007). DAVID Bioinformatics Resources: expanded annotation database and novel algorithms to better extract biology from large gene lists. Nucleic Acids Res..

