# Supplementary material for: Inferring pathway dysregulation in cancers from multiple types of omic data
Source: Genome Med. 2015 Jun 26;7(1):61. doi: 10.1186/s13073-015-0189-4 (PMC4499940; doi:10.1186/s13073-015-0189-4)
Supplement: Additional file 1: — Supplementary Methods, Figs. S1–S7, and Tables S1–S7. [file 13073_2015_189_MOESM1_ESM.pdf]

Inferring pathway dysregulation in cancers from multiple types of omic data

Supplementary Methods

Data sets

Simulated data. We used the *make_classification* function in the scikit-learn package[1] to simulate random, normally distributed data. This function introduces a subtle signal, with interdependence among the variables, as typically would be observed in omic data. The first simulated set contained data for 100 samples (2 classes) and 20,000 features. Such dimensions are commonly seen in omic data. Overall, 200 of the features contained signal; 25 features were “informative,” while the remaining 175 signal genes were “redundant” with these. For the initial analysis, 50% of the samples belonged in each class. In the second simulated data set, 90% of samples belonged to the first class, and 10% of samples belonged to the second class. With the latter data set, we aimed to simulate the common scenario in omic analyses where there is a strong class imbalance. We also generated a gene set database that contained 2300 gene sets, which ranged in size between 25 and 300 genes (in increments of 25). The genes for each gene set were randomly selected from the full simulated data set. Half of the gene sets were deliberately selected to contain no signal genes. The remaining gene sets contained a mix of signal and non-signal genes; the number of signal genes in these gene sets ranged between 5 and 50 (in increments of 5). All code that we used to generate this data set (and to perform all simulation analyses described in the paper) are available from https://bitbucket.org/srp33/gsoa.

Gene sets. Gene sets used as input for GSOA, GAGE, GSEA, GSAA and GSAAseqSP were downloaded from the Molecular Signatures Database[2]. These files contain gene sets established from experimentally generated data or manual curation, aggregated from existing databases, such as KEGG, Biocarta, Reactome, and the Pathway Interaction Database (PID)[3–6]. Gene set files used for the p53 (*s2.symbols.gct)* and gender analysis (*c1.symbols.gct* and *c2.symbols.gct)* were downloaded from the GSEA datasets page (http://www.broadinstitute.org/gsea/datasets.jsp). We used the same gene set files that were used in the original GSEA paper[7] to enable a more direct comparison between GSOA and GSEA

results. 1,320 gene sets from the C2: canonical pathways collection (*c2.cp.v4.0.symbols.gmt)* were used for the TCGA HER2 and uterine serous carcinoma analyses. 3402 experimentally derived gene sets from the C2: chemical and genetic perturbations collection (*c2.cgp.v4.0.symbols.gmt*) were used for the RAS analysis.

Microarray data . The p53 and Gender microarray datasets were downloaded from the GSEA webpage (http://www.broadinstitute.org/gsea/datasets.jsp). The p53 dataset, originally presented in Subramanian et al.[7] contains pre-normalized mRNA expression levels from the NCI-60 cell lines (17 wild type, 33 mutant). These were measured using Affymetrix HGU95Av2 chips, and contain 12,625 probe sets. The gender data set contains pre-normalized mRNA expression levels from lymphoblastoid cell lines (15 male, 17 female). No additional normalization or filtering steps were used. For our endometrial carcinoma MYC pathway validations, we used a microarray data set, GSE24537, obtained from the NCBI's Gene Expression Omnibus[8, 9]. As additional filtering, we removed probes that did not map to genes, and removed the bottom 30% expressing genes, resulting in 25,299 genes for the GSOA analysis.

TCGA data. We used "Level 3" data for the TCGA analyses. RNA-Sequencing, microarray, somatic mutation, copy number variation, and clinical data were downloaded from The Cancer Genome Atlas (TCGA) Pan-Cancer Initiative Project (PANCAN12) (https://www.synapse.org/#!Synapse:syn300013)[10]. One gene, "SLC24E2" was removed from all TCGA RNA-sequencing analyses because it was duplicated. We also excluded any variable that contained at least one missing value across the samples for a given data type. Subtype characteristics were obtained from TCGA clinical files. Endometrial clinical data was downloaded from (https://tcga-data.nci.nih.gov/docs/publications/ucec_2013/). We used Python and R scripts to reformat the data according to GSOA's specifications. We performed no additional preprocessing, other than the mean-centering and scaling procedure implemented within GSOA.

Parameters used for each analysis

GSOA, GSAA, and GSAAseqSP were executed at the command-line[11, 12]. GSEA was executed using the desktop tool for that algorithm [7]. GAGE was executed in R (3.1.2) and

Bioconductor (3.0) [13]. Our code repository provides scripts and parameters that were used to execute these analyses.

All GSOA analyses presented in this paper were performed with 1000 random iterations and 5-fold cross validation. For GSEA, GSAA, and GAGE, we used default parameters. For the p53 and Gender microarray analyses, we used the previously published results for GSEA, which excluded genes sets smaller than 10. For GAGE, the "microarray" and "test gene sets in both directions" features were used. For the lung adenocarcinoma (LUAD) RNA-Sequencing data set, we compared samples with a mutation in *HRAS, NRAS,* or *KRAS* to samples that did not have a mutation in these genes[14]. The "RNA-Sequencing" feature was used with GAGE, and "*GSEApreranked"* was used for GSEA. To maintain consistency, no gene sets were filtered for these analyses. For the breast cancer analysis, we compared HER2 positive samples against all other BRCA samples (luminal A, luminal B, basal, and normal)[15]. For the endometrial cancer analysis, we compared samples with serous histology against all other endometrial cancer samples (non-serous). Samples with mixed serous/non-serous histology were excluded from the analysis. The number of samples for each class and omic type is listed in Table 1.

Supplementary Figures

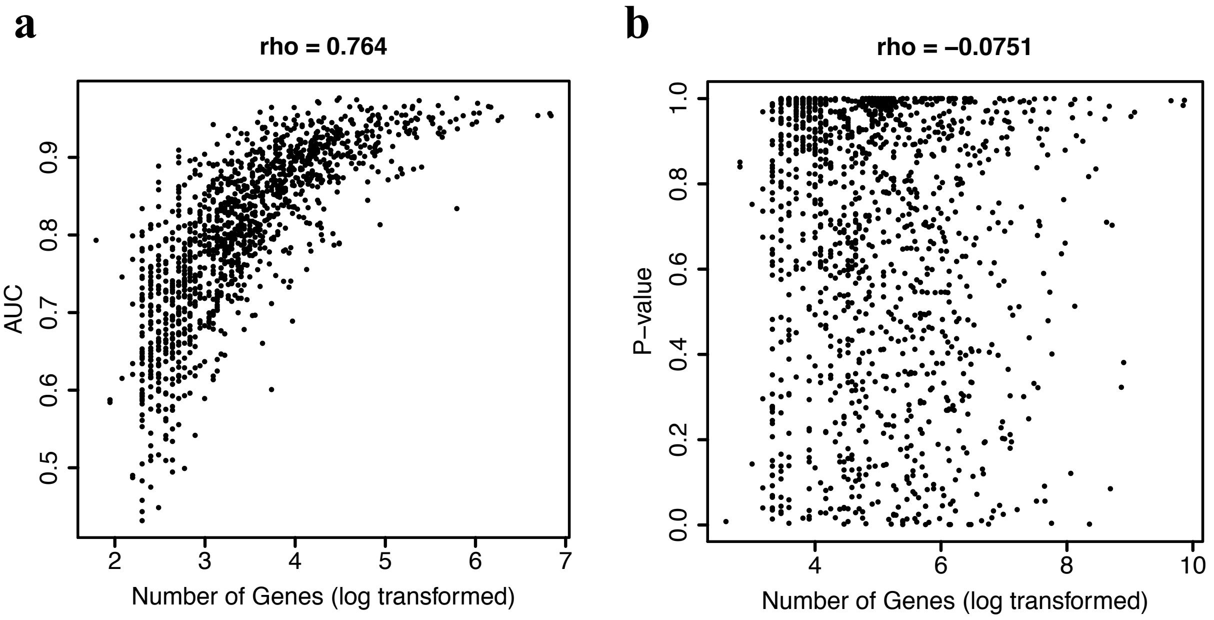

Supplementary Figure 1. Correlation between number of genes in a pathway and GSOA output values. We used GSOA to compare $HER2^+$ breast cancer samples against $HER2^-$ samples from TCGA. The natural log of the number of genes in each gene set was correlated with **A)** AUC values, but not with **B)** p-values. Spearman correlation coefficients were used to quantify similarity in ranks between the values.

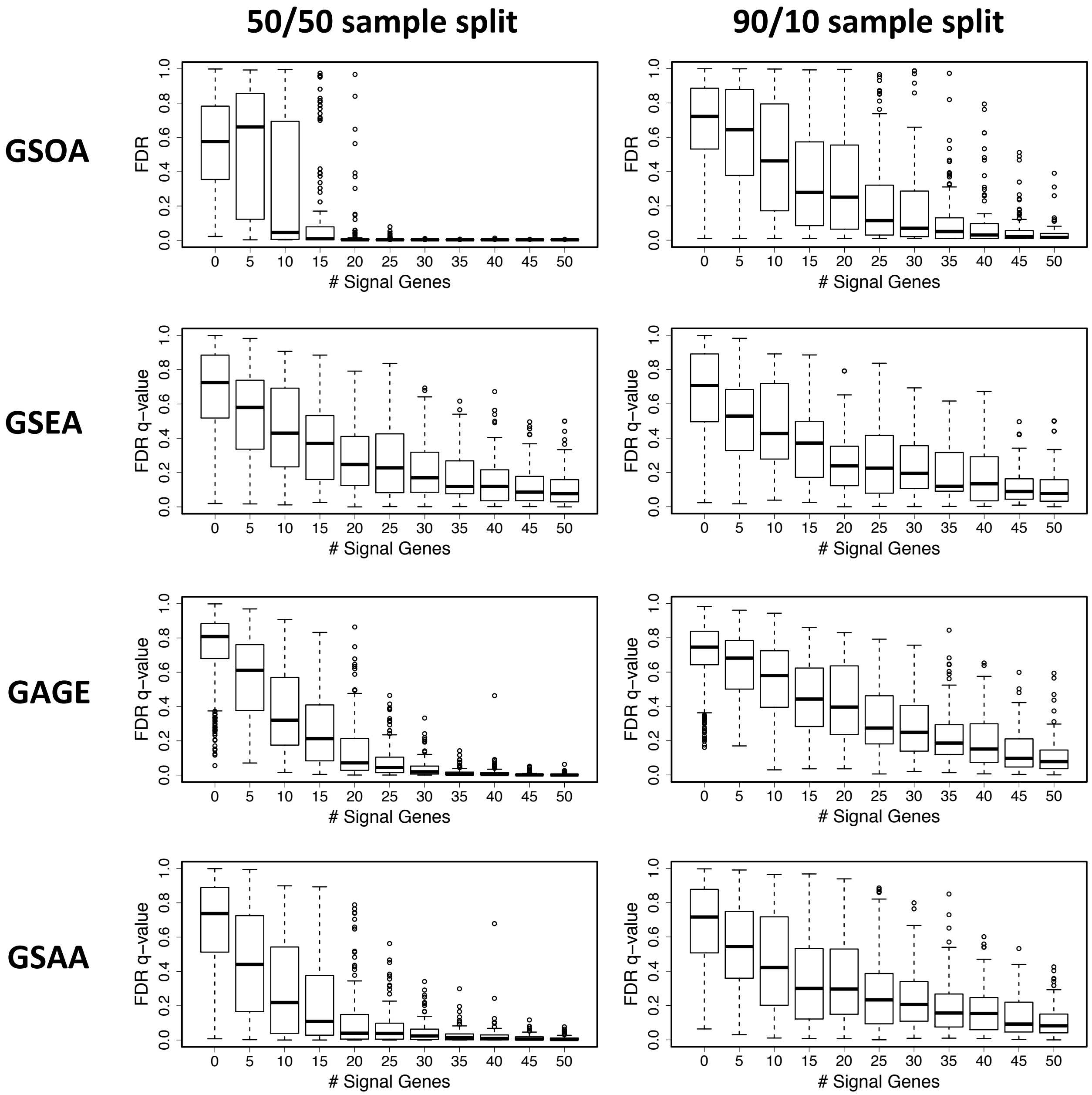

Supplementary Figure 2. FDR values from the simulation analysis for all methods using balanced (50/50 split) and unbalanced (90/10 split) class numbers.

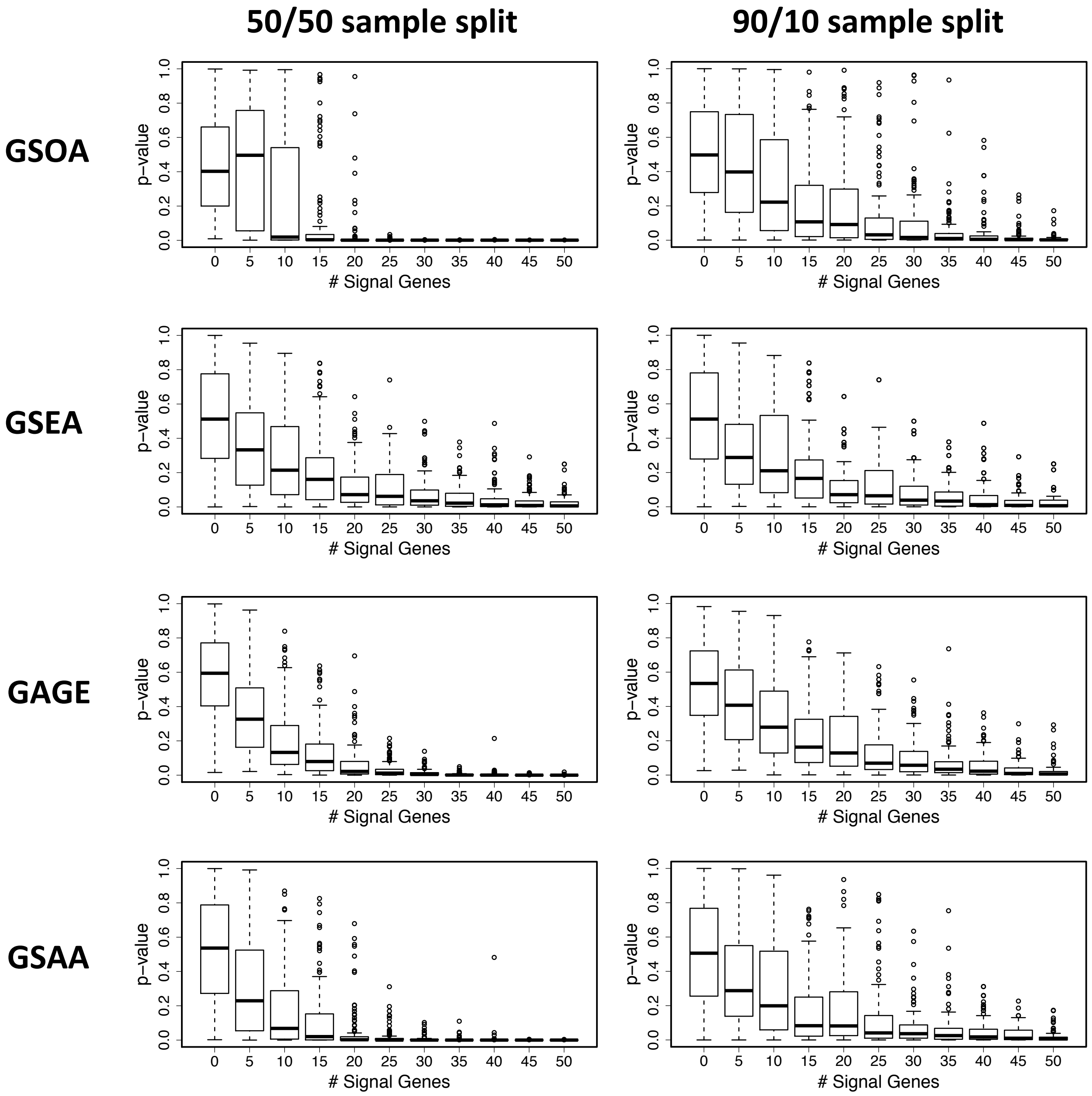

Supplementary Figure 3. p-values from the simulation analysis for all methods using balanced (50/50 split) and unbalanced (90/10 split) class numbers.

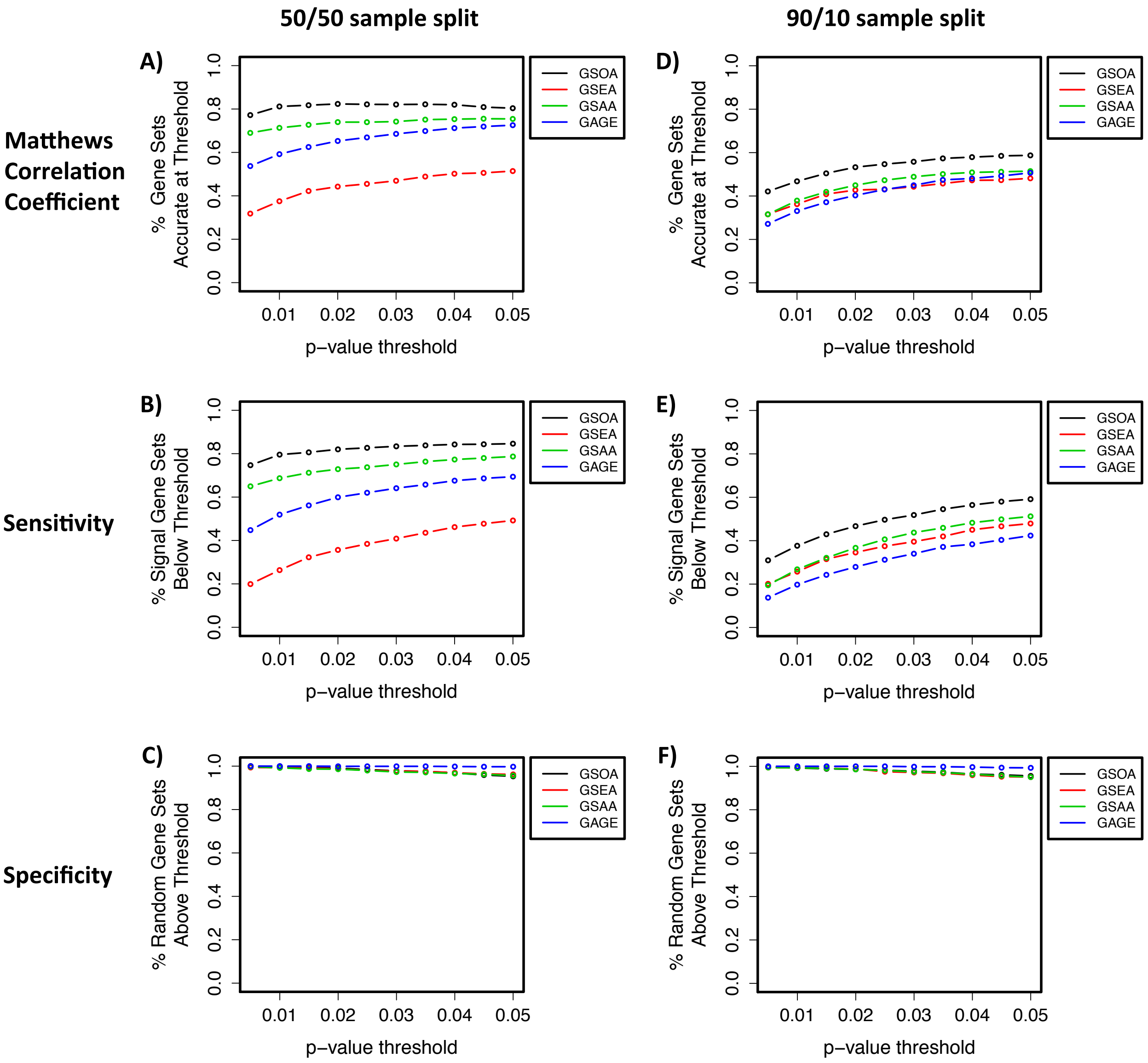

Supplementary Figure 4. Results of cross-algorithm comparisons on simulated data. We compared GSOA against other methods using simulated data that contained interdependence among variables. For various p-value thresholds, we calculated the proportion of simulated gene sets containing signal that were considered significant and the proportion of gene sets containing only random data that were not considered significant. The left panel contains results for balanced data (50/50 sample split); the right panel contains results for unbalanced data (90/10 sample split).

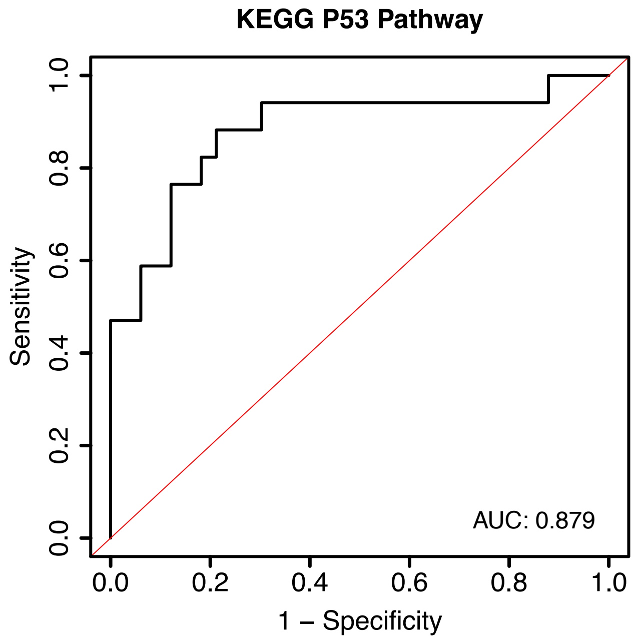

Supplementary Figure 5. ROC curve for the KEGG p53 pathway gene set from the p53 mutant vs. wild-type GSOA microarray anaylsis.

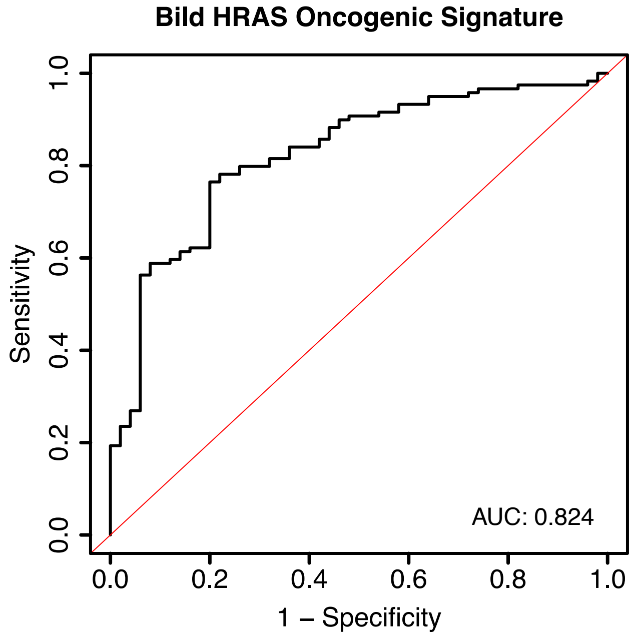

Supplementary Figure 6. ROC curve for the Bild HRAS oncogenic signature gene set from the GSOA RAS mutation anaylsis in lung adenocarcinoma.

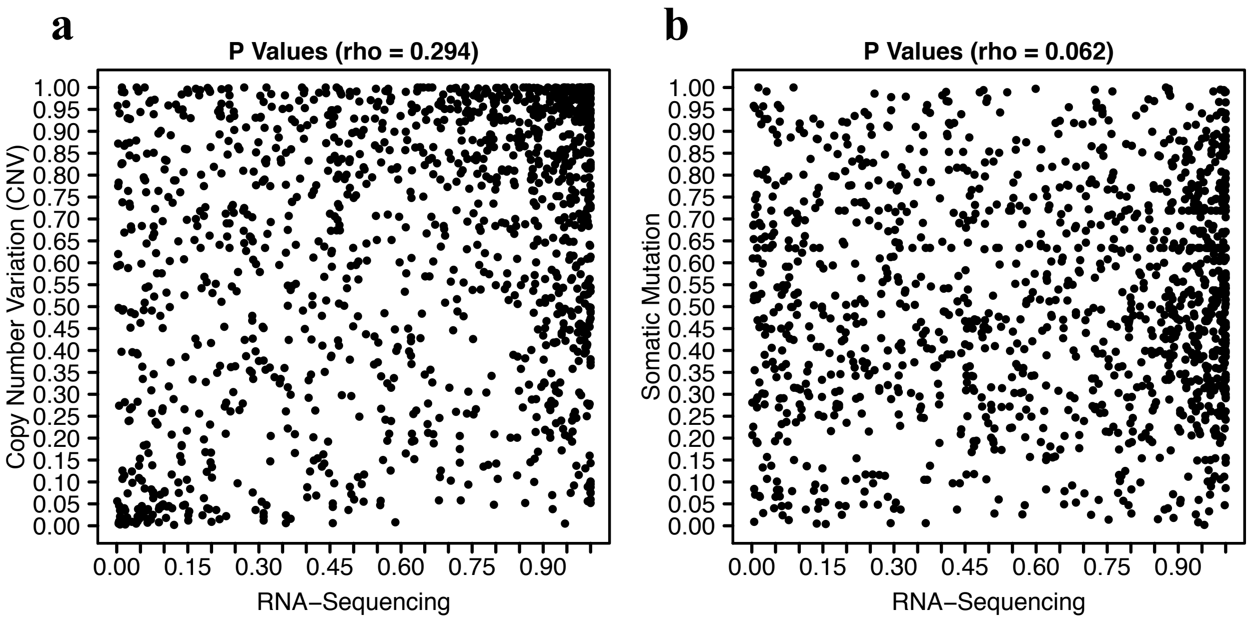

Supplementary Figure 7. Correlations between different omic types using GSOA produced p-values. GSOA was applied to TCGA data for copy-number variations, somatic mutations and RNA-Sequencing technologies. HER2-postive breast cancer samples were compared against HER2-negative breast cancer samples. GSOA output values were not highly correlative between **A)** copy-number variations and RNA-Sequencing levels or between **B)** somatic mutations and RNA-Sequencing levels.

Supplementary Tables

Supplementary Table 1. Performance metrics for simulated data analysis for FDR thresholds that are more or less stringent. These results were obtained using the balanced data (50/50 sample split). MCC = Matthews Correlation Coefficient.

A) FDR threshold = 0.05

	# True Positives	# True Negatives	# False Positives	# False Negatives	MCC
GSOA	946	1139	11	204	0.82
GSEA	213	1143	7	937	0.30
GSAA	703	1142	8	447	0.65
GAGE	634	1150	0	516	0.62

B) FDR threshold = 0.20

	# True Positives	# True Negatives	# False Positives	# False Negatives	MCC
GSOA	989	1021	129	161	0.75
GSEA	574	1107	43	576	0.52
GSAA	911	1076	74	239	0.74
GAGE	839	1144	6	311	0.75

Supplementary Table 2. Pathway-based comparison of lung adenocarcinoma samples based on RAS mutation status in TCGA lung adenocarcinoma RNA-Sequencing data. The Bild HRAS oncogenic signature was identified as significant among 3402 gene sets.

	Bild HRAS oncogenic signature			
Method	# of sig. gene sets	rank	p-val	FDR
**GSOA**	154/3402	28	0.009	0.972
**GSEA**	674/3402	1	0.000	0.004
**GAGE**	456/3402	68	0.000	0.019
**GSAAseqSP**	169/3402	512	0.177	1

Supplementary Table 3. Top 20 gene sets predicted by GSOA using a single SVM classifier for all multiomic data in HER2+ vs. HER2- TCGA samples. RNA-sequencing, somatic mutation, and CNV data were analyzed. Pathways related to ERBB signaling are in bold.

C2 canonical gene sets (MSigDB)	p-val	FDR
Reactome Nuclear Receptor Transcription Pathway	0.001	0.330
**KEGG Pathways In Cancer**	**0.001**	**0.330**
KEGG Bladder Cancer	0.001	0.330
**Reactome PI3K Events In ERBB2 Signaling**	0.002	0.330
**Reactome Signaling by FGFR**	**0.002**	**0.330**
**Reactome PI3K Cascade**	**0.002**	**0.330**
**Reactome Signaling by ERBB2**	0.002	0.330
**Reactome Downstream Signaling of Activated FGFR**	**0.002**	**0.330**
KEGG Pancreatic Cancer	0.003	0.440
PID MYC Repress Pathway	0.004	0.528
PID EPHB Fwd Pathway	0.006	0.660
PID FAK Pathway	0.007	0.660
Reactome Signaling by SCF KIT	0.007	0.660
**Reactome Down Regulation of ERBB2 ERBB3 Signaling**	0.007	0.660
**PID ERBB2 ERBB3 Pathway**	0.008	0.660
Reactome Downstream Signal Transduction	0.008	0.660
KEGG Prostate Cancer	0.011	0.776
**PID ERBB4 Pathway**	**0.012**	**0.776**
Biocarta IL2RB Pathway	0.013	0.776

Supplementary Table 4. **Top 20 gene sets predicted by GSOA using a rank-based method to aggregate evidence across multiple omic types for the HER2+ vs. HER2- comparisons.** RNA-sequencing, somatic mutation, and CNV data were analyzed, and pathways with a known association to ERBB signaling are in bold.

C2 canonical gene sets (MSigDB)	rank p-val	FDR
**Reactome PI3K Events In ERBB2 Signaling**	0.002	0.395
Reactome Signaling By PDGF	0.002	0.395
**Reactome Signaling By ERBB2**	0.002	0.395
ST Integrin Signaling Pathway	0.003	0.395
KEGG Bladder Cancer	0.003	0.395
KEGG Pancreatic Cancer	0.003	0.395
Reactome Downstream Signal Transduction	0.004	0.395
Reactome Mitotic G1 G1 S Phases	0.004	0.395
Reactome CDT1 Association With The CDC6 ORC Origin Complex	0.004	0.395
Reactome G1 S Transition	0.004	0.395
PID ERA Genomic Pathway	0.004	0.395
Reactome M G1 Transition	0.004	0.395
Reactome Downstream Signaling Events Of B-Cell Receptor BCR	0.005	0.395
**PID ERBB2 ERBB3 pathway**	0.005	0.395
Reactome ORC1 Removal From Chromatin	0.005	0.395
**PID ERBB4 Pathway**	0.005	0.395
Reactome Signaling By The B-Cell Receptor BCR	0.005	0.395
Reactome Assembly Of The Pre-Replicative Complex	0.006	0.395
PID E2F Pathway	0.007	0.395
Biocarta TOB1 Pathway	0.007	0.395

Supplementary Table 5. **Top 50 gene sets predicted by GSOA using a rank-based method to aggregate evidence for all omic data in USC vs. UEC TCGA samples.** RNA-sequencing, somatic mutation, and CNV data were analyzed, and gene sets with a known association to endometrial cancer are in bold.

C2 canonical gene sets (MSigDB)	rank p-val	FDR
**KEGG Phosphatidylinositol Signaling System**	**0.002**	**0.420**
KEGG Tight Junction	0.003	0.420
Reactome PI Metabolism	0.003	0.420
**KEGG Pathways In Cancer**	**0.004**	**0.420**
Biocarta Chemical Pathway	0.004	0.420
**Reactome PI3K Events In ERBB2 Signaling**	**0.004**	**0.420**
KEGG Prostate Cancer	0.004	0.420
KEGG Chronic Myeloid Leukemia	0.004	0.420
**SA PTEN Pathway**	**0.004**	**0.420**
Biocarta MET Pathway	0.005	0.420
KEGG Non Small Cell Lung Cancer	0.005	0.420
PID A6B1 A6B4 Integrin Pathway	0.005	0.420
KEGG Melanoma	0.005	0.420
Reactome Phospholipid Metabolism	0.006	0.420
KEGG Basal Cell Carcinoma	0.006	0.420
**Reactome ERK MAPK Targets**	**0.006**	**0.420**
KEGG Pancreatic Cancer	0.006	0.420
KEGG Melanogenesis	0.007	0.420
**PID P53 Downstream Pathway**	**0.007**	**0.420**
KEGG Small Cell Lung Cancer	0.007	0.420
**Biocarta P53 Pathway**	**0.007**	**0.420**
Reactome Nuclear Events Kinase And Transcription Factor Activation	0.007	0.420
KEGG WNT Signaling Pathway	0.008	0.420
KEGG Apoptosis	0.008	0.420
PID CXCR4 Pathway	0.008	0.420
KEGG Inositol Phosphate Metabolism	0.008	0.420
**Pid Myc Pathway**	**0.009**	**0.420**
Sig PIP3 Signaling In Cardiac Myoctes	0.009	0.420
Reactome Cell Cell Communication	0.009	0.420
KEGG Glioma	0.009	0.420
KEGG Bladder Cancer	0.010	0.420
**KEGG ERBB Signaling Pathway**	**0.010**	**0.420**
Reactome Amine Compound Slc Transporters	0.010	0.423
KEGG Leukocyte Transendothelial Migration	0.010	0.423
PID BCR5 Pathway	0.011	0.430
Reactome CTLA4 Inhibitory Signaling	0.011	0.432
St T Cell Signal Transduction	0.012	0.446
**Reactome PI3K Events In ERBB4 Signaling**	**0.013**	**0.473**
Reactome Na Cl Dependent Neurotransmitter Transporters	0.014	0.473
Reactome Intrinsic Pathway For Apoptosis	0.014	0.473
SA G1 And S Phases	0.014	0.473
St Integrin Signaling Pathway	0.015	0.473
**PID PI3KCI pathway**	**0.016**	**0.479**
Reactome Semaphorin Interactions	0.017	0.503
**Biocarta HER2 Pathway**	**0.017**	**0.505**
Reactome Cell Surface Interactions At The Vascular Wall	0.018	0.510
PID ER Nongenomic Pathway	0.018	0.510
**KEGG Endometrial Cancer**	**0.018**	**0.511**
Biocarta CHREBP2 Pathway	0.019	0.519

Supplementary Table 6. **Top 50 gene sets predicted by GSOA using a single SVM classifier for all omic data in USC vs. UEC TCGA samples.** RNA-sequencing, somatic mutation, and CNV data were analyzed, and gene sets with a known association to endometrial cancer are in bold.

C2 canonical gene sets (MSigDB)	p-val	FDR
**KEGG Pathways In Cancer**	**0.001**	**0.528**
KEGG Small Cell Lung Cancer	0.001	0.528
**Reactome PI3K Events In ERBB2 Signaling**	**0.002**	**0.528**
Biocarta MET Pathway	0.002	0.528
**PID IL2 PI3K Pathway**	**0.002**	**0.528**
**Reactome PI3K Events In ERBB4 Signaling**	**0.003**	**0.660**
Reactome Sema3a Plexin Repulsion Signaling By Inhibiting Integrin Adhesion	0.004	0.660
Reactome Signal Regulatory Protein SIRP Family Interactions	0.004	0.660
WNT Signaling	0.005	0.733
KEGG Apoptosis	0.008	1.000
KEGG Sphingolipid Metabolism	0.009	1.000
PID Trail Pathway	0.013	1.000
**KEGG Endometrial Cancer**	**0.013**	1.000
PID Nephrin NEPH1 Pathway	0.013	1.000
KEGG Prostate Cancer	0.013	1.000
**PID ERBB4 Pathway**	**0.013**	1.000
KEGG B-Cell Receptor Signaling Pathway	0.015	1.000
**Biocarta BARR MAPK Pathway**	**0.019**	1.000
Reactome PI Metabolism	0.019	1.000
Biocarta ACH Pathway	0.019	1.000
Biocarta EIF Pathway	0.019	1.000
**PID P53 Downstream Pathway**	**0.020**	1.000
PID Ceramide Pathway	0.022	1.000
Biocarta HCMV Pathway	0.023	1.000
Reactome Prostanoid Ligand Receptors	0.024	1.000
KEGG Axon Guidance	0.026	1.000
Reactome Cell Cell Communication	0.027	1.000
Reactome GAB1 Signalosome	0.028	1.000
**Reactome Signaling By ERBB4**	**0.029**	1.000
KEGG Melanogenesis	0.029	1.000
KEGG Tryptophan Metabolism	0.030	1.000
Biocarta PYK2 Pathway	0.031	1.000
PID MTOR4 pathway	0.031	1.000
PID E-cadherin Stabilization Pathway	0.033	1.000
PID FRA Pathway	0.033	1.000
PID Endothelin Pathway	0.036	1.000
**Reactome ERKs Are Inactivated**	**0.036**	1.000
PID P73 Pathway	0.037	1.000
**Reactome GRB2 Events In ERBB2 Signaling**	**0.037**	1.000
Biocarta Chemical Pathway	0.037	1.000
PID P38 Alpha Beta Down Stream Pathway	0.039	1.000
**Reactome PI3K Cascade**	**0.04**	1.000
KEGG WNT Signaling Pathway	0.04	1.000
Reactome IL1 Signaling	0.04	1.000
Reactome Nephrin Interactions	0.04	1.000
KEGG Acute Myeloid Leukemia	0.04	1.000
Reactome Regulatory RNA Pathways	0.04	1.000
Reactome Phospholipid Metabolism	0.041	1.000
**Reactome Signaling By FGFR In Disease**	**0.042**	1.000

Supplementary Table 7. Genes with significant somatic mutation or expression differences between USC and ESC in the Pathway Interaction Database *MYC pathway* gene set.

MYC Pathway	RNA-Seq Wilcox p-value	Direction	Mutated in USC
ACTL6A	5.0×10^{-5}	Up in USC	No
CDKN2A	1.9×10^{-16}	Up in USC	No
FBXW7	3.8×10^{-4}	Down in USC	32%
MYC	3.3×10^{-8}	Up in USC	No
PAK2	2.4×10^{-4}	Up in USC	No
PML	4.0×10^{-4}	Up in USC	No
RUVBL1	2.0×10^{-3}	Up in USC	No
SKP2	3.1×10^{-3}	Up in USC	No
SUPT7L	1.6×10^{-6}	Up in USC	No
TAF9	5.9×10^{-13}	Down in USC	No
TRRAP	3.8×10^{-4}	Up in USC	No

The Wilcoxon rank test was used for RNA-Sequencing data, and a Fisher's Exact test was used for somatic mutations.

References

1. Pedregosa F, Varoquaux G, Gramfort A, Michel V, Thirion B, Grisel O, Blondel M, Prettenhofer P, Weiss R, Dubourg V, Vanderplas J, Passos A, Cournapeau D, Brucher M, Perrot M, Duchesnay É: **Scikit-learn: Machine Learning in Python**. *J Mach Learn Res* 2011, **12**:2825–2830.
2. Liberzon A, Subramanian A, Pinchback R, Thorvaldsdóttir H, Tamayo P, Mesirov JP: **Molecular signatures database (MSigDB) 3.0.** *Bioinformatics* 2011, **27**:1739–40.
3. Kanehisa M, Goto S, Kawashima S, Okuno Y, Hattori M: **The KEGG resource for deciphering the genome.** *Nucleic Acids Res* 2004, **32**(Database issue):D277–80.
4. Nishimura D: **BioCarta**. *Biotech Softw Internet Rep* 2001, **2**:117–120.
5. Croft D, Mundo AF, Haw R, Milacic M, Weiser J, Wu G, Caudy M, Garapati P, Gillespie M, Kamdar MR, Jassal B, Jupe S, Matthews L, May B, Palatnik S, Rothfels K, Shamovsky V, Song H, Williams M, Birney E, Hermjakob H, Stein L, D'Eustachio P: **The Reactome pathway knowledgebase.** *Nucleic Acids Res* 2014, **42**(Database issue):D472–7.
6. Schaefer CF, Anthony K, Krupa S, Buchoff J, Day M, Hannay T, Buetow KH: **PID: the Pathway Interaction Database.** *Nucleic Acids Res* 2009, **37**(Database issue):D674–9.
7. Subramanian A, Tamayo P, Mootha VK, Mukherjee S, Ebert BL, Gillette MA, Paulovich A, Pomeroy SL, Golub TR, Lander ES, Mesirov JP: **Gene set enrichment analysis: a knowledge-based approach for interpreting genome-wide expression profiles.** *Proc Natl Acad Sci U S A* 2005, **102**:15545–50.
8. Barrett T, Wilhite SE, Ledoux P, Evangelista C, Kim IF, Tomashevsky M, Marshall KA, Phillippy KH, Sherman PM, Holko M, Yefanov A, Lee H, Zhang N, Robertson CL, Serova N, Davis S, Soboleva A: **NCBI GEO: archive for functional genomics data sets--update.** *Nucleic Acids Res* 2013, **41**(Database issue):D991–5.
9. Mhawech-Fauceglia P, Wang D, Kesterson J, Syriac S, Clark K, Frederick PJ, Lele S, Liu S: **Gene expression profiles in stage I uterine serous carcinoma in comparison to grade 3 and grade 1 stage I endometrioid adenocarcinoma.** *PLoS One* 2011, **6**:e18066.
10. Weinstein JN, Collisson EA, Mills GB, Shaw KRM, Ozenberger BA, Ellrott K, Shmulevich I, Sander C, Stuart JM: **The Cancer Genome Atlas Pan-Cancer analysis project.** *Nat Genet* 2013, **45**:1113–20.
11. Xiong Q, Ancona N, Hauser ER, Mukherjee S, Furey TS: **Integrating genetic and gene expression evidence into genome-wide association analysis of gene sets.** *Genome Res* 2012, **22**:386–97.
12. Xiong Q, Mukherjee S, Furey TS: **GSAASeqSP: a toolset for gene set association analysis of RNA-Seq data.** *Sci Rep* 2014, **4**:6347.
13. Luo W, Friedman MS, Shedden K, Hankenson KD, Woolf PJ: **GAGE: generally applicable gene set enrichment for pathway analysis.** *BMC Bioinformatics* 2009, **10**:161.
14. Collisson EA, Campbell JD, Brooks AN, Berger AH, Lee W, Chmielecki J, Beer DG, Cope L, Creighton CJ, Danilova L, Ding L, Getz G, Hammerman PS, Neil Hayes D, Hernandez B,

Herman JG, Heymach J V., Jurisica I, Kucherlapati R, Kwiatkowski D, Ladanyi M, Robertson G, Schultz N, Shen R, Sinha R, Sougnez C, Tsao M-S, Travis WD, Weinstein JN, Wigle DA, et al.: **Comprehensive molecular profiling of lung adenocarcinoma**. *Nature* 2014, **511**:543–550.
15. **Comprehensive molecular portraits of human breast tumours**. *Nature* 2012, **490**:61–70.
